# Meditation: A Promising Approach for Alleviating Chronic Pain

**DOI:** 10.7759/cureus.49244

**Published:** 2023-11-22

**Authors:** Akshay Dubey, Parikshit A Muley

**Affiliations:** 1 Physiology, Datta Meghe Medical College, Datta Meghe Institute of Higher Education and Research (DU), Nagpur, IND

**Keywords:** pain management, yoga, meditation, mindfulness, chronic pain

## Abstract

Chronic pain is a complex and pervasive health issue that significantly impacts the lives of millions. Various cultures have been practicing meditation for thousands of years, and it has been shown that it has many positive effects on mental and physical health. The impact of meditation on chronic pain is encouraging as it will form the base for future advancements. Meditation can improve the body's natural pain-relieving processes, lower stress levels, and boost body awareness. Patients can select from a variety of meditation techniques and include them in their treatment plans in a way that suits them best. Although it may not be a permanent solution, meditation can give patients a useful tool for managing their pain. In both clinical and experimental contexts, mindfulness meditation has been shown to lessen pain dramatically. Meditation may be used to manage chronic pain, which has several benefits, including pain alleviation, stress reduction, improved sleep, and general health. The research study provides insights into various kinds of meditation practices as well as the scientific basis of the mechanisms that are mentioned in the literature for the management of pain. Meditation practices have been scientifically shown in numerous randomized controlled studies to reduce pain intensity, enhance pain tolerance, and improve quality of life in those with chronic pain. Although more research is necessary to fully understand the mechanisms underlying the practice, the encouraging evidence is currently available.

## Introduction and background

Chronic pain, a pervasive and debilitating condition affecting millions worldwide, transcends mere physical discomfort to infiltrate every aspect of an individual's life. Its complicated nature intertwines physical suffering with emotional distress, often leading to a cascade of psychological and social challenges [1]. The first to describe this was Melzack's early writings, which suggested sensory, emotional, and cognitive-evaluative components to the pain experience [2].

Pain may be acute or chronic, depending upon its longevity. The pain that lasts for more than three months is referred to as chronic pain, while the acute pain gets healed within a month usually [3]. Chronic pain is also described as "pain that lasts longer than the anticipated time of healing." Chronic pain is distinct. The body still aches years after the incident occurred. Chronic pain is any form of pain that lasts between three and six months or more. Chronic pain may have a detrimental effect on everyday life and mental health [4].

In addition to the body, the brain and spinal cord can also be the source of chronic pain. It might be challenging to treat. According to epidemiological research, 8-11.2% of individuals worldwide suffer from persistent, widespread pain [5]. Chronic pain can vary in many different forms and is quite debilitating. Neuropathic, nociceptive, and neoplastic pain are a few types of chronic pain. It is linked to a decline in quality of life as well as an overall decrease in physical and emotional function [6]. The relevance of the psychological, social, and environmental elements of chronic pain is one of the important yet occasionally disregarded factors. Additionally, depression and addiction to painkillers are two psychiatric conditions that frequently coexist with chronic pain, complicating treatment. Understanding the most likely neurobiological mechanism(s) causing the current pain and how they may influence options for treatment is also frequently undervalued [6]. Ten percent of the world's population deals with chronic pain [7]. Credible estimates show that the prevalence of chronic pain is closer to 20-25% in each country and area [8]. Although there are few global estimates of the prevalence of chronic pain, the WHO has estimated that up to one in 10 adult patients worldwide receives a new diagnosis of chronic pain every year [7]. In addition to its frequency and incidence, the severity of the pain and the extent of any accompanying impairment are important criteria in characterizing the burden of chronic pain. As meditation helps in managing the chronic pain disorder and it is widely familiar to people across the world, it can be used for the treatment of chronic pain [9]. The objective of this article is to provide an information of chronic pain, its multifaceted nature, its prevalence, and the potential role of meditation in its management.

## Review

Meditation and its approach

One can acquire a state of calmness and mental clarity by meditating, which entails focusing one's attention on a particular object, sound, or concept. It has been a component of several cultures and faiths throughout the world for countless years. Movement meditation, mantra meditation, visualization meditation, and mindfulness meditation are just a few of the many different types of meditation. Developing a state of stillness, alertness, and inner tranquility is the aim of meditation. Chronic pain victims usually practice mindfulness meditation. Based on age-old Eastern meditation practices, mindfulness promotes an attentional attitude of dispassionate observation. Being open, inquisitive, and receptive to the situation as it develops defines it [10].

At the most elementary level, which is to transcend oneself, meditation is something people practice in part for the benefit of themselves. Usually, people identify with their minds, their bodies, and their thoughts and emotions. Thus, the thinking mind and the feeling body turn into a prison that most people live throughout their lives. By doing meditation, it is possible to transcend its limited identity and transform the mind and body into a place you can enter and exit rather than a place you are confined to [11].

People may experience pleasure without a desire and pain without suffering through meditation. Even though it may seem little, there is a crucial difference between pain and suffering. One of the main benefits of meditation for treating chronic pain is its ability to reduce stress levels. Giving an experience a lot of attention, maintaining sensory clarity, and being calm are the key characteristics of a spiritual path based on mindfulness [12]. Focusing attention on what is most important to people requires concentration. The ability to distinguish the elements of an experience and perceive their underlying essence is known as sensory clarity. Whether they are expanding, shrinking, or staying stationary, these components can continue functioning normally. Equanimity is the full absence of any disruption of our senses' normal course. In other words, one can try to be alert, accurate, and forgiving in every conversation [13].

Benefits

Meditation may be used to manage chronic pain, and there are several benefits to doing so [1]. Meditation can help to lessen stress and worry, which are usually associated with chronic pain. Due to their continuing discomfort and limitations on their daily activities, patients with chronic pain may feel more anxious. Meditation can aid in lowering these stress levels by promoting calmness and relaxation [14].

The quality of sleep, which is usually affected in those with chronic pain, can also be improved by meditation. Sleep is essential for healing, but chronic pain can make it difficult for the body to get the rest it needs. People can better manage their chronic pain by relaxing and getting a better night's sleep with the aid of meditation. Thirdly, meditation can raise one's standard of living [15]. Chronic pain can make it difficult to carry out everyday tasks, which can result in social isolation and diminished pleasure in life. Meditation can boost mood, increase social support, and improve general well-being when it is used to manage chronic pain [16].

Incorporation

It might be difficult to include meditation in the treatment of chronic pain, especially for people who have never done so. Meditation can be started with brief periods. It can be started with five-minute meditation sessions and, as one gets more accustomed to the practice, progressively be lengthened [17]. Choose a comfortable posture that supports one's body to sit or lie down comfortably. For back, neck, or leg support, utilize pillows or cushions. As one breathes in and out, pay attention to one's breath. If one's thoughts stray, gently refocus them on one's breathing [18]. The easiest way of meditating for a beginner is to just focus on their breathing pattern as it is an involuntary process one just has to observe when the air gets in and out of the lungs; while doing so, one gets to concentrate on something and the brain recognizes a rhythm and its focus gets shifted from unnecessary thoughts and it feels like a soothing experience to get out of the worries and just to be involved in something that is breathing [19].

Meditation techniques

A couple of meditation techniques listed below may help manage chronic pain. Mindfulness meditation is the practice of concentrating on the present moment while accepting any thoughts or sensations that arise without judgment [20]. Mantras are words or phrases that are repeated while meditating [21]. Exercises that promote relaxation and focus, such as yoga or tai chi, are used in movement meditation [22]. Visualization meditation uses mental pictures to promote calmness and reduce stress. The practice of mindfulness meditation entails concentrating people's attention on the current moment without passing judgment on it. This method can assist people in being more conscious of their body's feelings, including pain [23].

Practicing mindfulness meditation can help patients with chronic pain feel better and have less discomfort overall [24]. In loving-kindness meditation, people focus on thinking kind, optimistic thoughts for themselves and other people [25]. This routine helps lessen negative emotions like resentment, rage, and hostility that can make pain worse. Practicing loving-kindness meditation can help those with chronic low back pain experience less pain, sadness, and anxiety [26]. During body scan meditation, people gradually direct their attention to various regions of their body while taking note of any feelings they may be feeling [26]. This method can assist people in being more conscious of their body's responses to discomfort. Body scan meditation can lessen pain intensity and enhance the quality of life in those with persistent low back pain [27]. Transcendental meditation uses a mantra or a word or phrase that is repeatedly chanted to assist the focus of the mind and encourage relaxation [28]. In patients with chronic pain, this technique has been shown to lessen their level of discomfort, anxiety, and depression [29]. Yoga meditation is a combination of breathing exercises and physical postures with meditation [30]. This method aids with relaxation and deduction in stress while enhancing flexibility and strength as well. People with chronic low back pain have benefited from yoga meditation by having less pain intensity, and their overall health has also become better [31]. Table 1 shows some of the meditation techniques commonly used in chronic pain management. Each technique offers unique approaches to help individuals better cope with and reduce the impact of chronic pain on their lives.

**Table 1:** Meditation techniques for chronic pain.

Meditation technique	Description
Mindfulness meditation	Focuses on being fully present in the moment, observing pain without judgment, and promoting self-acceptance. It encourages non-reactive awareness to reduce suffering [20].
Guided imagery meditation	Involves creating calming mental images to distract from pain, induce relaxation, and promote a sense of well-being. Participants visualize soothing scenes or experiences [23].
Body scan meditation	A systematic process of directing attention to each part of the body, often starting from the toes and moving up, to increase body awareness and release tension or discomfort [27].
Transcendental meditation	A mantra-based practice that involves silently repeating a specific word or phrase to quiet the mind and attain a deep state of relaxation, potentially reducing pain perception [28].
Loving-kindness meditation	Focused on cultivating feelings of compassion and kindness, it can improve emotional well-being and reduce pain-related emotional suffering [25].
Zen meditation (Zazen)	Involves seated meditation with a focus on breath and posture, emphasizing a detached, non-judgmental awareness that can help individuals cope with chronic pain more effectively [1].
Vipassana meditation	A form of insight meditation, it encourages the observation of bodily sensations, thoughts, and emotions, promoting a deeper understanding of pain and its impermanence [26].
Yoga meditation	Yoga meditation is a combination of breathing exercises and physical postures with meditation [30].

Mindfulness and its role

Having a focused state, well mental health, and judgment-free state is known as mindfulness [32]. It's possible to effectively manage chronic pain by practicing mindfulness; through mindfulness, chronic pain can be therapeutically managed. The intricacy of chronic pain is influenced by physical, psychological, and cognitive factors. It has been shown that mindfulness-based treatments help reduce the intensity and suffering associated with chronic pain [33]. People who regularly practice mindfulness learn to accept others' suffering without judging it. This could minimize the agony that chronic pain commonly causes in people's minds. Furthermore, mindfulness can help individuals take more control over their pain, which can reduce anxiety [34].

Research has shown that mindfulness-based therapies are beneficial in treating chronic pain. Mindfulness-based interventions were shown to help lower pain intensity, pain interference, and mental distress related to chronic pain in a meta-analysis of randomized controlled trials (RCTs) [35]. Furthermore, a study found that practicing mindfulness can improve healthy people's pain tolerance indicating that mindfulness may have a pain-relieving effect [36].

The science behind meditation

Even though the research on the advantages of meditation in treating chronic pain is promising, more study is still required to fully comprehend the mechanisms underlying it. According to the study, mindfulness meditation reduces pain by activating the orbitofrontal cortex (OFC) and anterior cingulate cortex [37]. According to the researchers, these brain areas are related to pain self-control. Additionally, mindfulness meditation inhibits the thalamus, which functions as a sort of gateway that regulates which sensory data is allowed to enter higher brain centers [1].

Neurotransmitter Changes

Serotonin and norepinephrine, two neurotransmitters that are important in the processing of pain, may be released in different amounts as a result of meditation [38]. Studies have indicated that meditation can raise levels of serotonin and lower levels of norepinephrine, two chemicals linked to pain relief [39].

Regulatory Mechanism of Inflammatory Response

Usually, chronic pain and inflammation are linked [40]. Research has shown that meditation usually helps in reducing the body's inflammatory response. Studies have indicated that meditation can lower levels of inflammatory cytokines, including interleukin-6 (IL-6) and tumor necrosis factor-alpha (TNF-α), which are associated with chronic pain and inflammation [41].

Stress Reduction

Chronic pain has been linked to stress, anxiety, and depression. People with chronic pain have been shown to experience less stress and happier moods after practicing meditation [42]. Studies have shown that meditation helps lower cortisol levels, the main stress hormone, which can reduce pain [43].

Evidence from clinical studies

A growing body of clinical research supports the efficacy of meditation for chronic pain management. Numerous RCTs have demonstrated that meditation techniques can lead to reductions in pain intensity, increased pain tolerance, and improved quality of life in chronic pain patients. Meta-analyses of these studies suggest that meditation is a statistically significant and clinically relevant intervention for chronic pain [44].

Reductions in Pain Intensity

Multiple RCTs have consistently shown that meditation techniques can lead to significant reductions in pain intensity among individuals suffering from chronic pain conditions [45]. Participants who engage in meditation-based interventions often report experiencing less severe pain, which can have a substantial impact on their overall well-being. These reductions in pain intensity are particularly valuable because they provide an alternative to or reduction in the use of pain-relief medications, which can have adverse side effects and potential long-term risks, such as addiction [46].

Increased Pain Tolerance

Meditation has also been associated with increased pain tolerance, allowing individuals to endure pain for longer durations without experiencing the same levels of distress [47]. This effect can be particularly beneficial for individuals with chronic pain conditions, as it empowers them to engage in activities and tasks they may have previously avoided due to the fear of exacerbating their pain. Increased pain tolerance can lead to improved functionality and a better quality of life [48].

Improved Quality of Life

Chronic pain has a profound impact on an individual's quality of life, affecting physical, emotional, and social aspects. Clinical studies consistently report that meditation interventions improve the overall quality of life for chronic pain patients [46]. Meditation helps individuals develop coping strategies, reduce anxiety and depression associated with chronic pain, and enhance their emotional well-being. Moreover, the improvements in pain intensity and pain tolerance contribute to a more fulfilling and functional life [49].

Meta-Analyses and Statistical Significance

Meta-analyses, which combine and analyze the results from multiple individual RCTs, have been conducted to assess the collective impact of meditation on chronic pain [50]. These analyses consistently demonstrate that meditation is a statistically significant and clinically relevant intervention for chronic pain. In other words, the overall effect of meditation on pain reduction is not simply due to chance but represents a meaningful and reproducible improvement. This statistical significance adds weight to the argument for the incorporation of meditation into pain management strategies [51].

Complementary and Holistic Approach

Meditation is often considered a complementary and holistic approach to chronic pain management. It addresses not only the physical aspects of pain but also the psychological and emotional dimensions [52]. This holistic approach aligns with the biopsychosocial model of pain management, which recognizes that chronic pain is influenced by physical, psychological, and social factors. Meditation can empower individuals to manage the psychological and emotional aspects of pain, helping them to break the cycle of pain-related distress and suffering [53].

Future directions for research on meditation and chronic pain

Even though the evidence for meditation's benefits in treating chronic pain is encouraging, more research is needed to fully understand the mechanisms underlying it [40]. Future research can look at how different meditation techniques affect chronic pain, the optimal meditation techniques for reducing pain frequency and duration, and the long-term benefits of meditation for the management of chronic pain. According to recent research, mindfulness meditation may benefit those who suffer from chronic pain by lowering their suffering. Future studies should investigate the effects of mindfulness-based treatments on the spatiotemporal dynamics, brain activity patterns, and connectivity in chronic pain patients using a range of methods, including functional magnetic resonance imaging (fMRI) and electroencephalogram (EEG). To evaluate the effectiveness of treatments for chronic pain, biomarker research is necessary due to the limitations of self-reported outcomes [41]. In the future, there might be advanced studies that can help in determining the number of effective sessions required for reducing chronic pain, and people can have specific types of meditation techniques for helping manage chronic pain.

## Conclusions

Meditation can be used to manage chronic pain, which has several benefits, including pain alleviation, stress reduction, improved sleep, and general health. Although more research is necessary to fully understand the mechanisms underlying the practice, the encouraging evidence is currently available. Meditating as part of a patient's therapy regimen can help them take control of their symptoms and improve the quality of their lives. The incorporation of meditation into pain management can acknowledge the interconnectedness of the physical, psychological, and social aspects of chronic pain. Moreover, it provides individuals with an empowering and non-pharmacological means to manage their pain. It is essential to recognize that the effectiveness of different meditation techniques may vary from person to person and the choice of the most suitable method should be individualized. The future of chronic pain management could involve a more integrative and holistic approach, combining meditation with traditional treatments, to optimize patient outcomes.
